# Tooth Loss and Socioeconomic Inequalities in Disability and Mortality: A Large-scale Prospective Cohort Study in Japan

**DOI:** 10.2188/jea.JE20250127

**Published:** 2026-07-05

**Authors:** Yusuke Matsuyama, Richard G. Watt, Jun Aida

**Affiliations:** 1 Department of Dental Public Health, Institute of Science Tokyo, Tokyo, Japan; 2 Department of Epidemiology and Public Health, University College London, London, United Kingdom

**Keywords:** epidemiology, inequalities, dentistry

## Abstract

**Background:**

Socioeconomic inequalities in disability-free life span have been widening. We evaluated the mediating role of multiple modifiable risk factors, including tooth loss, on socioeconomic inequalities in disability onset and mortality among Japanese older adults.

**Methods:**

This prospective cohort study utilized data from the Japan Gerontological Evaluation Study, targeting adults aged ≥65 years. The 2013 baseline questionnaire survey participants were followed until 2022 (*n* = 48,474; median follow-up, 9.0 years). Time-varying mediators were also assessed in questionnaire surveys in 2016 and 2019. Discrete-time survival analysis estimated the association of socioeconomic status (SES)—a standardized principal component score incorporating household income, wealth, and years of education—with disability or mortality onset. The Karlson–Holm–Breen method decomposed total effects into pathways through 11 mediators, including tooth loss and major risk factors for disability and mortality.

**Results:**

During the follow-up, 29.1% became disabled or died. Compared to the highest SES group, the lowest SES quartile group exhibited a hazard ratio of 1.26 (95% confidence interval [CI], 1.19–1.34) for disability or mortality. Tooth loss exhibited the second largest indirect effect (proportion mediated 12.4%; 95% CI, 8.0–17.2), following moderate depression (16.0%; 95% CI, 11.7–21.5). Tooth loss exhibited the strongest association with SES, attributing to the large indirect effect.

**Conclusion:**

The findings suggest that tackling inequalities in tooth loss may be an effective way to reduce socioeconomic inequalities in a disability-free life span.

## INTRODUCTION

Socioeconomic circumstances in which people live significantly influence their health outcomes, leading individuals with lower socioeconomic status (SES) to experience an earlier onset of disability and mortality.^1^^–^^3^ Disability-free life span, defined by the occurrence of either disability or mortality as the endpoint, serves as a key measure for assessing population health and health inequalities.^4^^,^^5^ While the average duration of disability-free life span has been extended, inequalities have increased globally over the past 3 decades.^6^ This trend underscores the necessity of public health strategies to reduce health inequalities. Clarifying intermediate modifiable risk factors and evaluating the extent of their contribution to socioeconomic inequalities in disability-free life spans would provide valuable insights for determining factors to be addressed.

Previous studies have estimated the contribution of several modifiable risk factors to health inequalities, mainly focusing on tobacco smoking, alcohol consumption, physical activity, and nutritional factors.^2^^,^^7^^–^^9^ These factors account for 9–36% of the inequalities in disability among older adults in the United States.^9^ In addition, a systematic review has reported that these factors jointly contribute 20–26% to all-cause mortality and 16–33% to cardiovascular disorders.^2^ A multi-cohort study by Laine et al incorporated additional health conditions, including body mass index, hypertension, diabetes, and coronary artery disease, as potential mediators. Their findings indicated that those factors jointly mediated the impact of SES on all-cause mortality by 38% for men and 34% for women.^7^ The clustering of the unhealthy behavioral factors in people with lower SES, rather than an inherent vulnerability among them, primarily explained the inequalities in mortality.^8^

Despite its significant burden on population health, oral health has been neglected in global health policy and research priorities.^10^ Notably, oral diseases are among the most prevalent diseases worldwide, affecting more than 3.5 billion people,^11^ and oral diseases substantially impact general health outcomes, including disability and mortality.^12^^,^^13^ The Global Burden of Disease 2019 Study elucidated that oral diseases ranked the 10th highest leading cause of total years lived with disability for adults aged 70 years or above.^14^ As a result of the high prevalence, tooth loss demonstrated the greatest population-attributable fraction of mortality among older Japanese men.^15^ The stark health inequalities in oral diseases across life stages^16^^–^^18^ also suggest that tooth loss may play a pivotal role among modifiable factors mediating health inequalities in the onset of disability and mortality. In fact, a study reported that tooth loss partially mediated the association between household income, one of the indicators of SES, and dementia onset among Japanese older adults.^19^ The preventability of oral diseases through the adoption of evidence-based policies and practices further underscores the importance of focusing on oral health in research to reduce health inequalities.

To our knowledge, no studies have investigated and compared the mediating role of oral conditions in health inequalities related to disability and mortality along with other modifiable risk factors. The present study aimed to examine the mediating role of tooth loss, compared with other modifiable risk factors, in socioeconomic inequalities in disability and mortality in older Japanese adults.

## METHODS

### Study participants

This prospective cohort study uses the Japan Gerontological Evaluation Study (JAGES) data, targeting adults aged ≥65 years.^20^ The present study utilized the data from the questionnaire surveys in 2013, 2016, and 2019 linked with information on the date of disability onset and mortality by 2022 obtained from the local municipalities’ government registry (follow-up rate, 90.7%; median follow-up, 9.0 years). The modifiable risk factors (ie, mediators) were assessed in 2013, 2016, and 2019 to incorporate their time-varying nature. The final analysis sample comprised 48,474 individuals with 416,779 person-year observations (see eMaterial 1, eFigure 1, eFigure 2, eFigure 3, eTable 1, eTable 2, eTable 3, eTable 4, and eTable 5). This study followed the STROBE reporting guidelines.

### Outcome variable: disability and mortality

The outcome was defined as the first occurrence of either disability onset or all-cause mortality obtained from the municipal and national databases. The disability onset was determined based on the certification for long-term care insurance (LTCI), a Japanese social insurance system that helps frail older individuals.^21^ The LTCI has seven care levels determined by capacity for activities of daily living and cognitive function (requiring help 1–2 and long-term care level 1–5). Certification at long-term care levels 2–5 (indicating a severe care level) represents a dependency on basic activities of daily living.^22^ It has previously been used to estimate healthy life expectancy in Japan.^23^ Therefore, we considered long-term care levels 2–5 to be the onset of disability.

### Exposure variable: socioeconomic status at baseline

The exposure, SES at baseline, was assessed with equivalized household income including pension, equivalized household wealth, and respondents’ years of education using the questionnaires in the 2013 survey. We incorporated these multiple factors to account for the complex interplay of various socioeconomic circumstances across the life course that shape health inequalities in older adults.^24^ Principal component analysis (PCA) was performed to incorporate the three dimensions of SES into a single scale.^25^ The predicted factor score was reversed and standardized to present the estimates in terms of increased risk. Further details on variable assessment and the PCA analysis are provided in eMaterial 1.

### Mediator variables: health conditions and behaviors in 2013, 2016, and 2019

The potential mediators were selected based on a previous study reporting the ten leading causes of years of life with disability in older people.^14^ The JAGES survey items relevant to those factors were selected. The time-varying information on the mediators was incorporated from the 2013, 2016, and 2019 surveys. The mediators included the number of natural teeth (having <20 natural teeth or not)^26^; moderate depression (the Geriatric Depression Scale 15 score ≥5)^27^; three-item subjective cognitive complaints^28^; diabetes; stroke; eye disease; ear disease; falling experience in the past year; smoking status; drinking status; and walking time. The information on diabetes, stroke, eye disease, and ear disease was assessed by asking participants to choose diseases or health conditions under treatment or having long-term effects. The details of the measurements of mediators and their categorizations are described in Table 1 and eMaterial 1.

**Table 1.** Demographic characteristics of the study participants; multiple imputation applied

		SES score^a^ (quartile categories)			

	Overall	Highest	Higher	Lower	Lowest
	*n* = 48,474	25.1%	24.9%	25.2%	24.8%
	*n* (%)^b^	*n* (%)^b^	*n* (%)^b^	*n* (%)^b^	*n* (%)^b^
**Endpoint of follow-up**					
Became disabled	9,110 (18.8%)	1,729 (14.2%)	2,038 (16.9%)	2,440 (20.0%)	2,904 (24.1%)
Died	9,530 (19.7%)	1,912 (15.7%)	2,193 (18.2%)	2,550 (20.9%)	2,876 (23.9%)
Became disabled or died^c^	14,118 (29.1%)	2,749 (22.6%)	3,225 (26.7%)	3,764 (30.9%)	4,380 (36.4%)
**SES in 2013**					
Equivalent income (million JPY)^d^	2.2 (1.4)	3.7 (1.7)	2.4 (0.9)	1.8 (0.8)	1.0 (0.6)
Equivalent wealth (million JPY)^d^	13.0 (10.8)	24.7 (9.2)	15.9 (8.0)	8.0 (6.5)	3.3 (3.3)
Years of education^d^	10.3 (3.2)	13.1 (3.1)	10.8 (2.9)	9.6 (2.3)	7.8 (1.7)
**Baseline confounders in 2013**					
Age^d^	73.6 (6.1)	72.5 (5.8)	73.1 (5.9)	73.9 (6.1)	75.0 (6.2)
Gender					
Men	22,586 (46.6%)	6,324 (51.9%)	5,901 (48.9%)	5,517 (45.2%)	4,844 (40.3%)
Women	25,888 (53.4%)	5,861 (48.1%)	6,164 (51.1%)	6,682 (54.8%)	7,181 (59.7%)
Marital status					
Not married	12,819 (26.4%)	2,632 (21.6%)	2,500 (20.7%)	3,416 (28.0%)	4,270 (35.5%)
Married	35,655 (73.6%)	9,553 (78.4%)	9,565 (79.3%)	8,783 (72.0%)	7,754 (64.5%)
Self-rated health					
Very good/good	40,344 (83.2%)	10,746 (88.2%)	10,274 (85.2%)	9,968 (81.7%)	9,356 (77.8%)
Poor/very poor	8,130 (16.8%)	1,439 (11.8%)	1,791 (14.8%)	2,232 (18.3%)	2,669 (22.2%)
Functional limitation^e^					
No	20,920 (43.2%)	6,448 (52.9%)	5,595 (46.4%)	4,953 (40.6%)	3,924 (32.6%)
Yes	27,554 (56.8%)	5,737 (47.1%)	6,470 (53.6%)	7,246 (59.4%)	8,101 (67.4%)
**Mediators in 2013; *n* = 48,474**					
Having <20 teeth	23,788 (49.1%)	4,399 (36.1%)	5,306 (44.0%)	6,501 (53.3%)	7,581 (63.0%)
Moderate depression^f^	12,572 (25.9%)	1,917 (15.7%)	2,655 (22.0%)	3,492 (28.6%)	4,508 (37.5%)
Cognitive complaints^g^	16,959 (35.0%)	3,531 (29.0%)	3,881 (32.2%)	4,453 (36.5%)	5,094 (42.4%)
Diabetes	6,394 (13.2%)	1,539 (12.6%)	1,554 (12.9%)	1,616 (13.2%)	1,686 (14.0%)
Stroke	1,497 (3.1%)	309 (2.5%)	354 (2.9%)	408 (3.3%)	426 (3.5%)
Falling experience	10,946 (22.6%)	2,303 (18.9%)	2,511 (20.8%)	2,797 (22.9%)	3,335 (27.7%)
Eye disease	10,187 (21.0%)	2,410 (19.8%)	2,425 (20.1%)	2,577 (21.1%)	2,776 (23.1%)
Ear disease	3,033 (6.3%)	606 (5.0%)	688 (5.7%)	795 (6.5%)	944 (7.9%)
Current smoking	4,782 (9.9%)	1,064 (8.7%)	1,136 (9.4%)	1,294 (10.6%)	1,288 (10.7%)
Current drinking	17,358 (35.8%)	5,170 (42.4%)	4,536 (37.6%)	4,141 (33.9%)	3,511 (29.2%)
Less walking^h^	12,160 (25.1%)	2,434 (20.0%)	2,748 (22.8%)	3,224 (26.4%)	3,754 (31.2%)
**Mediators in 2016; *n* = 44,895**					
Having <20 teeth	22,931 (51.1%)	4,393 (38.2%)	5,250 (46.5%)	6,198 (55.2%)	7,090 (65.2%)
Moderate depression^f^	10,951 (24.4%)	1,724 (15.0%)	2,322 (20.6%)	3,039 (27.1%)	3,865 (35.5%)
Cognitive complaints^g^	16,548 (36.9%)	3,483 (30.3%)	3,878 (34.4%)	4,307 (38.3%)	4,880 (44.9%)
Diabetes	6,144 (13.7%)	1,546 (13.4%)	1,524 (13.5%)	1,543 (13.7%)	1,532 (14.1%)
Stroke	1,502 (3.3%)	319 (2.8%)	343 (3.0%)	380 (3.4%)	460 (4.2%)
Falling experience	11,657 (26.0%)	2,475 (21.5%)	2,652 (23.5%)	3,054 (27.2%)	3,477 (32.0%)
Eye disease	10,175 (22.7%)	2,415 (21.0%)	2,450 (21.7%)	2,545 (22.7%)	2,765 (25.4%)
Ear disease	2,983 (6.6%)	610 (5.3%)	683 (6.1%)	776 (6.9%)	914 (8.4%)
Current smoking	4,021 (9.0%)	937 (8.1%)	955 (8.5%)	1,061 (9.4%)	1,068 (9.8%)
Current drinking	15,589 (34.7%)	4,794 (41.7%)	4,123 (36.5%)	3,665 (32.6%)	3,008 (27.7%)
Less walking^h^	13,642 (30.4%)	2,824 (24.5%)	3,168 (28.1%)	3,574 (31.8%)	4,076 (37.5%)
**Mediators in 2019; *n* = 39,241**					
Having <20 teeth	20,527 (52.3%)	4,111 (39.6%)	4,812 (48.0%)	5,491 (56.5%)	6,113 (67.0%)
Moderate depression^f^	10,480 (26.7%)	1,770 (17.0%)	2,338 (23.3%)	2,876 (29.6%)	3,496 (38.3%)
Cognitive complaints^g^	14,864 (37.9%)	3,299 (31.8%)	3,559 (35.5%)	3,843 (39.6%)	4,163 (45.6%)
Diabetes	5,641 (14.4%)	1,490 (14.3%)	1,442 (14.4%)	1,369 (14.1%)	1,340 (14.7%)
Stroke	1,349 (3.4%)	331 (3.2%)	337 (3.4%)	347 (3.6%)	333 (3.7%)
Falling experience	10,700 (27.3%)	2,400 (23.1%)	2,524 (25.2%)	2,764 (28.5%)	3,013 (33.0%)
Eye disease	9,537 (24.3%)	2,395 (23.1%)	2,313 (23.1%)	2,402 (24.7%)	2,427 (26.6%)
Ear disease	3,139 (8.0%)	677 (6.5%)	738 (7.4%)	805 (8.3%)	918 (10.1%)
Current smoking	2,788 (7.1%)	678 (6.5%)	664 (6.6%)	724 (7.5%)	722 (7.9%)
Current drinking	12,909 (32.9%)	4,078 (39.3%)	3,443 (34.4%)	2,973 (30.6%)	2,415 (26.5%)
Less walking^h^	13,156 (33.5%)	2,883 (27.7%)	3,132 (31.3%)	3,430 (35.3%)	3,711 (40.7%)

SES, socioeconomic status.

^a^Derived from the first component of principal component analysis with equivalent income, equivalent wealth, and years of education.

^b^Means across imputed datasets are reported.

^c^Either of disability or all-cause mortality onset

^d^Values show mean and standard deviation.

^e^Assessed with the Tokyo Metropolitan Institute of Gerontology Index of Competence.

^f^Assessed with the Geriatric Depression Scale 15 score ≥5.

^g^Having subjective cognitive complaints ≥1.

^h^Walking <0.5 hour a day

### Baseline confounders

The following variables in the 2013 survey were included as the baseline confounders: age, gender, marital status, self-rated health, having any limitations in higher-level functional capacity.^29^ Residential municipality was also adjusted for, given that the outcome data were obtained from municipal registries.

### Statistical analysis

Discrete-time survival analysis was used to incorporate time-varying mediators. The data for discrete-time survival analysis were constructed in a long format, with one observation for every year of age in which the individual participated in the study. Information on mediators, each measured at three time points, was organized into a single column based on participant age to estimate the mediator-specific indirect effect during the follow-up period. Accordingly, individuals who developed the outcome before each follow-up survey do not have corresponding information on the variable from that survey.

eFigure 4 shows the directed acyclic graph. First, the association of SES with disability onset or mortality, adjusting for baseline confounders, was investigated. Second, the association was decomposed into indirect effects through each mediator and direct effects not through any of them using the Karlson–Holm–Breen (KHB) method.^30^ We selected the KHB method rather than the causal mediation framework because the latter required a substantial assumption of directional association among the mediators to disentangle the total indirect effects into path-specific ones. Third, we plotted the coefficients for the exposure-mediator association and that for mediator-outcome association from the KHB method, to evaluate whether the mediator-specific indirect effect was derived by the association between SES and mediator or that between mediator and outcome (see eMaterial 1). The variable of SES was grouped into quartile categories in the first analysis confirming social gradient, while it was standardized and used as a continuous variable in the KHB method.

Missing information on variables was imputed by multiple imputations with chained equations, generating ten imputed data sets for the main analysis (see eMaterial 1). Imputation diagnostics were evaluated by visual inspection of trace plots and by comparing distributions of the imputed and observed variables. Sensitivity analysis under the missing-not-at-random (MNAR) assumption using the delta method was performed.^31^^,^^32^

Sensitivity analyses with different cutoffs for mediator variables and complete case analysis were also performed. Sub-analyses with mortality, disability, and mild disability (LTCI certification for long-term care level 1 or higher) as separate outcomes were also performed. In addition, causal mediation analyses were conducted for the mediators that showed greater contributions in the KHB method. All analyses were performed using Stata MP version 18 software (Stata Corp, College Station, TX, USA).

### Ethics approval

The ethics committees at Nihon Fukushi University (13-14), Chiba University (No. 2493 and 3442), National Center for Geriatrics and Gerontology (No. 992 and 1274-2), Japan Agency for Gerontological Evaluation Study (No. 2019-01), and Tokyo Medical and Dental University (No. D2022-040) approved the present study. This study followed the guidelines of the Declaration of Helsinki.

## RESULTS

Table 1 describes the demographic characteristics of the study participants. The mean age at baseline was 73.6 years, and 46.6% were men. The lower SES groups had unhealthy mediator conditions than the higher SES groups. For example, in 2013, the prevalence of having <20 teeth in the highest, higher, lower, and lowest SES groups was 36.1%, 44.0%, 53.3%, and 63.0%, respectively. Among the mediators, tooth loss showed the most notable difference by SES groups (eg, 26.9 percentage-point differences between the highest and the lowest groups in 2013).

Table 2 reports the association between lower SES and the onset of disability or mortality estimated by discrete-time hazard models. Compared to the highest SES group, the second, third, and lowest SES quartile groups exhibited hazard ratios (HR) of 1.12 (95% confidence interval [CI], 1.06–1.18), 1.20 (95% CI, 1.14–1.27), and 1.26 (95% CI, 1.19–1.34) for the onset of disability or mortality after adjusting for the baseline confounders only, respectively. Complete case analysis showed similar results (eTable 6).

**Table 2.** Association between socioeconomic status and disability or all-cause mortality; *n* = 48,474 individuals; multiple imputation applied

	Incidence rate^a^	Crude	Adjusted^b^
		HR (95% CI)	HR (95% CI)
SES quartile categories			
Highest	27.8	Reference	Reference
Higher	33.6	1.22 (1.15–1.28)	1.12 (1.06–1.18)
Lower	39.7	1.45 (1.37–1.54)	1.20 (1.14–1.27)
Lowest	48.0	1.78 (1.69–1.88)	1.26 (1.19–1.34)

CI, confidence interval; HR, hazard ratio; SES, socioeconomic status.

^a^Incidence rate per 1,000 person-years

^b^Adjusted for age, gender, marital status, self-rated health, limitation in functional capacity, and residential municipality at the baseline.

**Figure 1.**
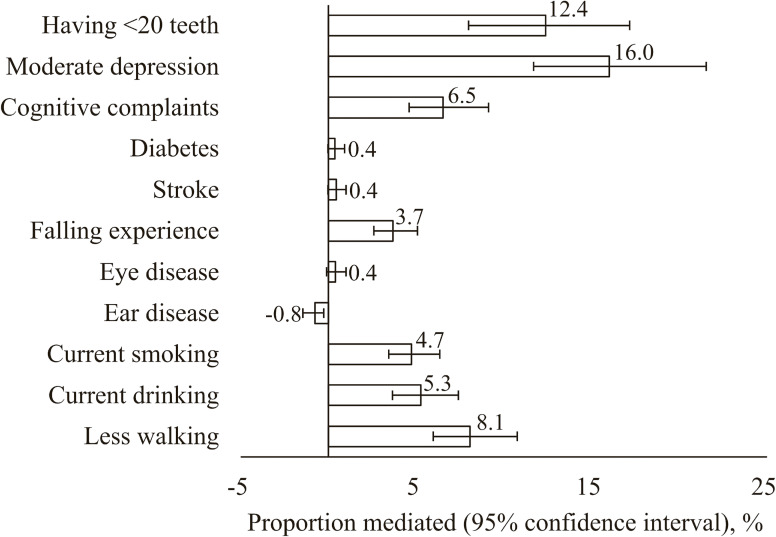
Proportion mediated for the association between socioeconomic status and disability or all-cause mortality; adjusted for age, gender, marital status, self-rated health, functional limitations, and residential municipality at the baseline; *n* = 48,474 individuals; multiple imputation applied

Table 3 presents the results of effect decomposition using the KHB method. The mediators significantly jointly mediated the association between SES and onset of disability and mortality (joint indirect effect HR 1.056; 95% CI, 1.049–1.063), accounting for 56.9% of total effects. Tooth loss demonstrated the largest indirect effect (HR 1.012; 95% CI, 1.008–1.016) for disability and mortality, following moderate depression (HR 1.015; 95% CI, 1.011–1.020). As depicted in Figure 1, tooth loss accounted for the second largest proportion of the association of SES with disability and mortality (12.4%; 95% CI, 8.0–17.2%), following depressive symptoms (16.0%; 95% CI, 11.7–21.5%). The sensitivity analyses under MNAR assumptions showed similar results (eTable 7). Complete case analysis showed a smaller mediated proportion than the imputed data, and tooth loss showed the largest mediation effect (eTable 8). The sensitivity analysis with different cutoffs for mediators showed that complete loss of natural teeth had moderate but significant indirect effects (eTable 9).

**Table 3.** Effect decomposition on the association of socioeconomic status with disability or all-cause mortality; *n* = 48,474 individuals; multiple imputation applied

	HR (95% CI)	*P*-value	PM (95% CI^d^)
Total effect	1.101 (1.079–1.124)	<0.001	—
Direct effect	1.042 (1.020–1.066)	<0.001	—
Joint indirect effect	1.056 (1.049–1.063)	<0.001	56.9 (45.5–71.7)
Each indirect effect			
Having <20 teeth	1.012 (1.008–1.016)	<0.001	12.4 (8.0–17.2)
Moderate depression^a^	1.015 (1.011–1.020)	<0.001	16.0 (11.7–21.5)
Cognitive complaints^b^	1.006 (1.004–1.008)	<0.001	6.5 (4.6–9.1)
Diabetes	1.000 (1.000–1.001)	0.129	0.4 (0.0–0.9)
Stroke	1.000 (1.000–1.001)	0.083	0.4 (0.0–1.0)
Falling experience	1.004 (1.003–1.005)	<0.001	3.7 (2.6–5.1)
Eye disease	1.000 (1.000–1.001)	0.195	0.4 (−0.1 to 1.0)
Ear disease	0.999 (0.999–1.000)	0.011	−0.8 (−1.5 to −0.3)
Current smoking	1.005 (1.003–1.006)	<0.001	4.7 (3.4–6.4)
Current drinking	1.005 (1.004–1.007)	<0.001	5.3 (3.7–7.4)
Less walking^c^	1.008 (1.006, 1.009)	<0.001	8.1 (6.0, 10.8)

CI, confidence interval; HR, hazard ratio; PM, proportion mediated.

Models were adjusted for age, gender, marital status, self-rated health, functional limitation, and residential municipality at the baseline.

^a^Assessed with the Geriatric Depression Scale 15 score ≥5.

^b^Having subjective cognitive complaints ≥1.

^c^Walking <0.5 hour a day

^d^95% CI were obtained using bootstrap with 200 resamples for each imputed dataset.

**Figure 2.**
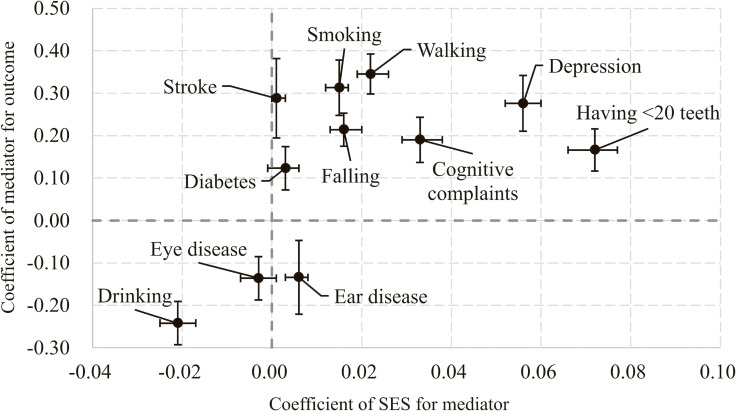
Plots for the mediator-outcome relationship against exposure-mediators relationship; adjusted for age, gender, marital status, self-rated health, functional limitations, and residential municipality at the baseline; *n* = 48,474 individuals; multiple imputation applied

Figure 2 depicts the estimates of the coefficients representing the association of SES with mediators (X-axis) and the association of mediators with disability and mortality (Y-axis). Among the mediators, tooth loss demonstrated the largest association with SES. For example, the estimates indicated that the prevalence of tooth loss increased by 7.2 percentage points per 1 standard deviation lower SES factor score. As for the mediator-outcome association, tooth loss was associated with disability and mortality with a moderate magnitude compared to other mediators.

The results of the sub-analysis with mortality, disability, and mild disability as separate outcomes are shown in eTable 10 and eTable 11. Similar associations of SES with these outcomes were observed (eTable 10). Moderate depression exhibited similar magnitudes of indirect effects for the separate outcomes, while tooth loss demonstrated a greater indirect effect for mortality than for disability (eTable 11). eTable 12 presents the results of the causal mediation analysis for tooth loss and depression. The proportions mediated by tooth loss in 2013, 2016, and 2019 ranged from 14.6% to 16.7% (model 1), which were comparable to those observed using the KHB method. However, the proportions were attenuated after adjusting for tooth loss measured in prior waves (model 2). The proportions mediated by moderate depression in 2013, 2016, and 2019 ranged from 5.1% to 32.7% (model 1), with effect sizes increasing in later waves. This pattern persisted even after adjusting for moderate depression measured in prior waves (model 2).

## DISCUSSION

The present study utilized a large-scale multiple-wave cohort study of older Japanese adults and found that a lower SES score incorporating the dimensions of income, wealth, and years of education was associated with disability onset and all-cause mortality. Among various traditional risk factors, tooth loss demonstrated the second largest indirect effect for the disability-free life span defined by the onset of either disability or mortality, mediating 12.4% of the association. Among the modifiable factors examined in this study, tooth loss exhibited the most substantial health inequality, while the association with disability onset or mortality was of moderate magnitude. Thus, the substantial mediating role of tooth loss among mediators was derived from its largest health inequalities.

The association of lower SES with disability onset and mortality has been well documented.^1^^–^^3^ For example, a multicohort study with 13 years of follow-up reported that people with low SES had 1.34 and 1.42 times higher mortality than their high SES counterparts, among men and women, respectively.^1^ A systematic review reported that smoking, alcohol drinking, and physical activity jointly contributed to health inequalities in mortality by 20–26%.^2^ The effect sizes of tooth loss in our study were comparable to those of major modifiable risk factors. Although direct comparisons of their review with our study are difficult due to different populations, measurements, and analytic approaches, those factors in our study showed smaller mediating effects than tooth loss. In addition, the relatively modest health inequalities in disability and mortality observed in our study may be explained by the universal healthcare system in Japan, the study population who have survived to 65 years of age or older.

Importantly, no studies have considered oral diseases in this context along with other modifiable factors. We found that tooth loss demonstrated a substantial contribution to inequalities in disability-free life span, defined by either disability onset or mortality. Although the sensitivity analysis using complete loss of natural teeth as a mediator indicated a smaller mediating effect, this result underestimated the impact of tooth loss by excluding the increased risk of disability onset and mortality among people with partial tooth loss.^12^ In the main analysis, we employed cut-off values that maximized the mediating effect of each mediator, and the result indicated that tooth loss had the second largest mediating effect. Furthermore, the causal mediation analysis for tooth loss showed that the mediating effect was attenuated after controlling for tooth loss measured in prior waves, suggesting that the cumulative impact of tooth loss plays an important role on shaping health inequalities in disability and mortality.

We found that tooth loss, and particularly inequalities in tooth loss, served as a significant determinant of health inequalities in disability-free life span. Previous studies have reported stark and persistent health inequalities in oral conditions.^16^^–^^18^ The harmful effect of poor oral conditions related to socioeconomic disadvantage accumulates throughout life, leading to significant inequalities in oral conditions in older age. Inflammation related to past periodontal disease, poor nutritional diet due to reduced masticatory function, and psychosocial and behavioral pathways, including reduced social interaction, which may link tooth loss and general health conditions.^33^^,^^34^ While few studies have quantitatively evaluated the pathways linking tooth loss with disability or mortality, previous research has shown that vegetable and fruit intake mediated 4.4–8.4% and social networks mediated 4.0–13.9% of the association between tooth loss and dementia in Japanese older adults.^33^ The indirect effect of tooth loss observed in the present study may also reflect the social functioning of older individuals. In addition, oral diseases serve as “a canary in the coalmine,” a marker of accumulated social disadvantage from early life,^35^ and thus they may strongly predict future onset of disability and mortality.

Oral diseases are largely preventable through evidence-based public health measures that address risk factors.^36^ Population strategies have the potential to effectively prevent oral diseases and their associated risk factors in people with lower SES.^37^^–^^39^ For example, universal dental health coverage can increase dental visits and reduce tooth loss in adults.^40^ Water fluoridation in residential areas at birth was associated with natural teeth retention in adulthood, with greater benefit for people with lower SES.^41^ Additionally, taxation on sugar-sweetened beverages in Mexico resulted in a higher reduction in the consumption of taxed beverages among low SES groups than high SES groups.^37^ While research is ongoing on what strategies effectively reduce oral health inequalities, there are compelling cases to support the benefit of population strategies. Such strategies have the potential to contribute to the reduction of oral health inequalities, which may lead to reducing inequalities in disability-free life span.

The strength of this study includes the large-scale cohort data of older Japanese adults for nine years with a high follow-up rate (90.7%). Mediators were assessed multiple times to incorporate the time-varying nature of modifiable risk factors. The participants’ SES was assessed with multiple dimensions, and the pathways underlying health inequalities in disability and mortality were disentangled according to specific modifiable risk factors. The findings provide valuable insights for public health policy to reduce health inequalities in disability-free life span.

This study also has limitations. First, the KHB method provides associational decomposition rather than causal estimates based on the counterfactual framework. The findings indicated that tooth loss significantly accounted for health inequalities in disability and mortality; however, it does not measure the extent to which interventions targeting tooth loss could potentially reduce these inequalities. Also, our approach does not consider the causal structure among mediators. The estimated indirect effect for each mediator is independent of other mediators, which may lead to an underestimation of mediator-specific indirect effects. Estimating sequential mediation effects requires accurate identification of the causal structure among mediators; however, with 11 mediators, this would involve substantial assumptions. Instead of relying on strong assumptions about causal structure, we estimated the lower bound of each path by controlling for the effects of the other mediators. In addition, the KHB method did not evaluate potential interaction effects between the mediator and SES on the outcome onset. Therefore, interaction effects were examined in the discrete-time survival analysis, and most of the results were not statistically significant (eTable 13).

Second, residual confounding may exist in the exposure-outcome, exposure-mediator, and mediator-outcome associations. While we have confirmed that results were robust to adjustment for denture use (eTable 14), other unmeasured confounders, for example, baseline dental caries and periodontitis, and systemic health conditions during follow-up, may have induced bias. Additionally, although we excluded participants with limitations in basic activities of daily living at baseline and adjusted for self-rated health and higher-level functional capacity limitations, reverse causation may still be present. Third, the SES score was derived from the first component of PCA, which explained 54% of the information from income, wealth, and years of education. The other two components did not increase the model performance in explaining the outcome (eTable 15). The first component of PCA would have sufficiently captured the aspects of SES associated with disability onset and mortality. Meanwhile, the PCA approach is not tailored to the study outcomes of disability and mortality. A hybrid approach that combines theory-driven selection with data-driven dimension reduction^42^ could generate an SES construct that is more predictive of these outcomes.

Fourth, some measurements lacked validation. For example, the binary responses measuring eye or ear disease have not been previously validated. By analyzing the subsample, we confirmed that having eye or ear disease was strongly associated with vision or hearing impairments (see eMaterial 1). The delays for LTCI certification may misalign with the disability onset timing. The self-reported tooth loss did not consider different reasons for tooth loss that can differentially impact disability. Also, measurement error may exist in self-reported tooth counts. The self-reported SES measures were not validated with objective measures; however, they were consistently associated with poor health. Fifth, some variables had a relatively higher proportion of missing information, particularly in the later waves of the questionnaire survey. We selected multiple imputation rather than excluding them to utilize their information on the outcome variable obtained from the municipality databases, and we have also confirmed that results were robust under several MNAR scenarios (eTable 7).

Sixth, the study was conducted in Japan, an egalitarian society with affordable healthcare under universal health coverage, compared to other high-income countries. The mediating effect of tooth loss may be different in other countries because of the high tooth retention rates in Japanese older adults, owing to affordable dental care.^43^ Seventh, the generalizability to the Japanese older population of the finding based on the participants in the 15 municipalities may be limited because, for example, JAGES participants were inclined to those with higher SES than the Japanese population.

In conclusion, the present study revealed that tooth loss had a substantial mediating role in the association between SES and disability onset and mortality among Japanese older adults. Tackling oral health inequalities may effectively reduce socioeconomic inequalities in disability-free life span. The findings support the need for oral health to be prioritized in future public health policies.

## SUPPLEMENTARY MATERIAL

eMaterial 1. Supplemental methods

eTable 1. Participation pattern

eFigure 1. Flowchart of the study participants

eFigure 2. Scree plot of eigenvalues from PCA analysis

eTable 2. Factor loadings for socioeconomic status (n = 48,474)

eFigure 3. Histogram of factor score for socioeconomic status

eTable 3. Association of having eye or ear disease with vision or hearing impairment: result from subsample responded to a particular version of the questionnaire in 2016 and 2019

eTable 4. Frequency of missing responses

eTable 5. Comparison of response, complete-case, and imputed samples

eFigure 4. Directed acyclic graph

eTable 6. Discrete-time survival analysis with complete case participants (n = 92,460 person-year observations from 11,311 individuals)

eTable 7. Effect decomposition analysis with missing not at random assumption

eTable 8. Effect decomposition analysis with complete case participants (n = 92,460 person-year observations from 11,311 individuals)

eTable 9. Effect decomposition analysis with different cutoffs. Different cutoffs were used for tooth loss (edentulous or having ≥1 teeth) and depression (having severe depression or not); multiple imputation applied

eTable 10. Discrete-time survival analysis with separate outcomes; n = 48,474 individuals; multiple imputation applied

eTable 11. Effect decomposition analysis with separate outcomes; n = 48,474 individuals; multiple imputation applied

eTable 12. Causal mediation analysis for tooth loss and depression

eTable 13. Test for interaction effects between socioeconomic status and mediators for disability or mortality; results from discrete-time survival analysis with multiple imputation

eTable 14. Effect decomposition analysis, further controlling for denture use as a covariate; n = 48,474 individuals; multiple imputation applied

eTable 15. Association between principal component factors and disability or mortality

## References

[1] Stringhini S, Carmeli C, Jokela M, ; LIFEPATH consortium. Socioeconomic status and the 25 × 25 risk factors as determinants of premature mortality: a multicohort study and meta-analysis of 1·7 million men and women. Lancet. 2017;389(10075):1229–1237. 10.1016/S0140-6736(16)32380-7 28159391 PMC5368415

[2] Petrovic D, de Mestral C, Bochud M, . The contribution of health behaviors to socioeconomic inequalities in health: a systematic review. Prev Med. 2018;113:15–31. 10.1016/j.ypmed.2018.05.003 29752959

[3] Head J, Chungkham HS, Hyde M, . Socioeconomic differences in healthy and disease-free life expectancy between ages 50 and 75: a multi-cohort study. Eur J Public Health. 2019;29(2):267–272. 10.1093/eurpub/cky215 30307554 PMC6426044

[4] Wood R, Sutton M, Clark D, McKeon A, Bain M. Measuring inequalities in health: the case for healthy life expectancy. J Epidemiol Community Health. 2006;60(12):1089–1092. 10.1136/jech.2005.044941 17108308 PMC2465513

[5] Kim YE, Jung YS, Ock M, Yoon SJ. A review of the types and characteristics of healthy life expectancy and methodological issues. J Prev Med Public Health. 2022;55(1):1–9. 10.3961/jpmph.21.580 35135043 PMC8841197

[6] Permanyer I, Villavicencio F, Trias-Llimós S. Healthy lifespan inequality: morbidity compression from a global perspective. Eur J Epidemiol. 2023;38(5):511–521. 10.1007/s10654-023-00989-3 37027116 PMC10080172

[7] Laine JE, Baltar VT, Stringhini S, . Reducing socio-economic inequalities in all-cause mortality: a counterfactual mediation approach. Int J Epidemiol. 2020;49(2):497–510. 10.1093/ije/dyz248 31855265 PMC7266549

[8] Puka K, Buckley C, Mulia N, Lasserre AM, Rehm J, Probst C. Educational attainment and lifestyle risk factors associated with all-cause mortality in the US. JAMA Health Forum. 2022;3(4):e220401. 10.1001/jamahealthforum.2022.0401 35445213 PMC8994133

[9] Shaw BA, McGeever K, Vasquez E, Agahi N, Fors S. Socioeconomic inequalities in health after age 50: are health risk behaviors to blame? Soc Sci Med. 2014;101:52–60. 10.1016/j.socscimed.2013.10.040 24560224 PMC3933820

[10] Watt RG, Daly B, Allison P, . Ending the neglect of global oral health: time for radical action. Lancet. 2019;394(10194):261–272. 10.1016/S0140-6736(19)31133-X 31327370

[11] Kassebaum NJ, Smith AGC, Bernabé E, ; GBD 2015 Oral Health Collaborators. Global, regional, and national prevalence, incidence, and disability-adjusted life years for oral conditions for 195 countries, 1990–2015: a systematic analysis for the global burden of diseases, injuries, and risk factors. J Dent Res. 2017;96(4):380–387. 10.1177/0022034517693566 28792274 PMC5912207

[12] Matsuyama Y, Aida J, Watt RG, . Dental status and compression of life expectancy with disability. J Dent Res. 2017;96(9):1006–1013. 10.1177/0022034517713166 28605598

[13] Matsuyama Y, Listl S, Jürges H, Watt RG, Aida J, Tsakos G. Causal effect of tooth loss on functional capacity among older adults in England. J Am Geriatr Soc. 2021;69(5):1319–1327. 10.1111/jgs.17021 33496349

[14] GBD 2019 Ageing Collaborators. Global, regional, and national burden of diseases and injuries for adults 70 years and older: systematic analysis for the Global Burden of Disease 2019 Study. BMJ. 2022;376:e068208. 10.1136/bmj-2021-068208 35273014 PMC9316948

[15] Nakazawa N, Kusama T, Cooray U, . Large contribution of oral status for death among modifiable risk factors in older adults: the JAGES prospective cohort study. J Gerontol A Biol Sci Med Sci. 2023;78(1):167–173. 10.1093/gerona/glac052 35231123

[16] Singh A, Peres MA, Watt RG. The relationship between income and oral health: a critical review. J Dent Res. 2019;98(8):853–860. 10.1177/0022034519849557 31091113

[17] Matsuyama Y, Tsakos G, Listl S, Aida J, Watt RG. Impact of dental diseases on quality-adjusted life expectancy in US adults. J Dent Res. 2019;98(5):510–516. 10.1177/0022034519833353 30849271

[18] Steele J, Shen J, Tsakos G, . The interplay between socioeconomic inequalities and clinical oral health. J Dent Res. 2015;94(1):19–26. 10.1177/0022034514553978 25344336

[19] Shimada S, Matsuyama Y, Aida J. Tooth loss explains income inequalities in dementia. J Dent. 2025;153:105518. 10.1016/j.jdent.2024.105518 39653269

[20] Kondo K, Rosenberg M; World Health Organization. Advancing Universal Health Coverage through Knowledge Translation for Healthy Ageing: Lessons Learnt from the Japan Gerontological Evaluation Study. World Health Organization; 2018. https://apps.who.int/iris/handle/10665/279010. Accessed 17.01.2023.

[21] Ministry of Health Labour and Welfare. Long-term care insurance in Japan. 2002. http://www.mhlw.go.jp/english/topics/elderly/care/index.html. Accessed 30.10.2024.

[22] Imahashi K, Kawagoe M, Eto F, Haga N. Clinical status and dependency of the elderly requiring long-term care in Japan. Tohoku J Exp Med. 2007;212(3):229–238. 10.1620/tjem.212.229 17592210

[23] Kataoka A, Fukui K, Sato T, . Geographical socioeconomic inequalities in healthy life expectancy in Japan, 2010–2014: an ecological study. Lancet Reg Health West Pac. 2021;14:100204. 10.1016/j.lanwpc.2021.100204 34527999 PMC8355904

[24] Spiers GF, Liddle JE, Stow D, . Measuring older people’s socioeconomic position: a scoping review of studies of self-rated health, health service and social care use. J Epidemiol Community Health. 2022;76(6):572–579. 10.1136/jech-2021-218265 35292509 PMC9118079

[25] Vyas S, Kumaranayake L. Constructing socio-economic status indices: how to use principal components analysis. Health Policy Plan. 2006;21(6):459–468. 10.1093/heapol/czl029 17030551

[26] Petersen PE, Baez RJ, World Health Organization. *Oral Health Surveys: Basic Methods*. 5th ed. World Health Organization; 2013. https://apps.who.int/iris/handle/10665/97035. Accessed 20.07.2023.

[27] Shin C, Park MH, Lee SH, . Usefulness of the 15-item geriatric depression scale (GDS-15) for classifying minor and major depressive disorders among community-dwelling elders. J Affect Disord. 2019;259:370–375. 10.1016/j.jad.2019.08.053 31470180

[28] Tomata Y, Sugiyama K, Kaiho Y, Sugawara Y, Hozawa A, Tsuji I. Predictive ability of a simple subjective memory complaints scale for incident dementia: evaluation of Japan’s national checklist, the “Kihon Checklist”. Geriatr Gerontol Int. 2017;17(9):1300–1305. 10.1111/ggi.12864 27506749

[29] Koyano W, Shibata H, Nakazato K, Haga H, Suyama Y. Measurement of competence: reliability and validity of the TMIG Index of Competence. Arch Gerontol Geriatr. 1991;13(2):103–116. 10.1016/0167-4943(91)90053-S 15374421

[30] Karlson KB, Holm A, Breen R. Comparing regression coefficients between same-sample nested models using logit and probit: a new method. Sociol Methodol. 2012;42(1):286–313. 10.1177/0081175012444861

[31] van Buuren S. *Flexible Imputation of Missing Data, Second Edition*. 2nd ed. Chapman and Hall/CRC; 2018. doi:10.1201/9780429492259. 10.1201/9780429492259

[32] Nguyen CD, Lee KJ, White IR, van Buuren S, Moreno-Betancur M. Sensitivity analysis for multivariable missing data using multiple imputation: a tutorial. *arXiv*. Preprint posted online 2025. doi:10.48550/ARXIV.2502.02892. 10.48550/ARXIV.2502.02892

[33] Kiuchi S, Cooray U, Kusama T, . Oral status and dementia onset: mediation of nutritional and social factors. J Dent Res. 2022;101(4):420–427. 10.1177/00220345211049399 34796750

[34] Tonsekar PP, Jiang SS, Yue G. Periodontal disease, tooth loss and dementia: Is there a link? A systematic review. Gerodontology. 2017;34(2):151–163. 10.1111/ger.12261 28168759

[35] Watt RG, Mathur MR, Aida J, Bönecker M, Venturelli R, Gansky SA. Oral health disparities in children: a canary in the coalmine? Pediatr Clin North Am. 2018;65(5):965–979. 10.1016/j.pcl.2018.05.006 30213357

[36] Peres MA, Macpherson LMD, Weyant RJ, . Oral diseases: a global public health challenge. Lancet. 2019;394(10194):249–260. 10.1016/S0140-6736(19)31146-8 31327369

[37] Colchero MA, Popkin BM, Rivera JA, Ng SW. Beverage purchases from stores in Mexico under the excise tax on sugar sweetened beverages: observational study. BMJ. 2016;352:h6704. 10.1136/bmj.h6704 26738745 PMC4986313

[38] Schwendicke F, Thomson WMM, Broadbent JMM, Stolpe M. Effects of taxing sugar-sweetened beverages on caries and treatment costs. J Dent Res. 2016;95(12):1327–1332. 10.1177/0022034516660278 27671690

[39] Hobbs M, Wade A, Jones P, . Area-level deprivation, childhood dental ambulatory sensitive hospitalizations and community water fluoridation: evidence from New Zealand. Int J Epidemiol. 2020;49(3):908–916. 10.1093/ije/dyaa043 32347945

[40] Raittio E, Suominen AL. Effects of universal oral healthcare coverage in an adult population: a long-term nationwide natural experiment. Community Dent Oral Epidemiol. 2023;51(5):908–917. 10.1111/cdoe.12785 36036466

[41] Neidell M, Herzog K, Glied S. The association between community water fluoridation and adult tooth loss. Am J Public Health. 2010;100(10):1980–1985. 10.2105/AJPH.2009.189555 20724674 PMC2936985

[42] Dao ATM, Do LG, Stormon N, Nguyen HV, Ha DH. Enhancing socioeconomic status prediction for cavities: a hybrid method. J Dent Res. 2025;104(9):947–954. 10.1177/00220345251324494 40102739 PMC12209541

[43] Aida J, Fukai K, Watt RG. Global neglect of dental coverage in universal health coverage systems and Japan’s broad coverage. Int Dent J. 2021;71(6):454–457. 10.1016/j.identj.2020.12.027 33618834 PMC9275350

