# Supplementary material for: Tooth Loss and Socioeconomic Inequalities in Disability and Mortality: A Large-scale Prospective Cohort Study in Japan
Source: J Epidemiol. 2026 Jul 5;36(7):233–40. doi: 10.2188/jea.JE20250127 (PMC13265305; doi:10.2188/jea.JE20250127)
Supplement: Supplementary file 1 [file je-36-233-s001.pdf]

eMaterial 1. Supplemental methods

Study participants

The flow chart of study participants is shown in eFigure 1. The 2013 baseline questionnaire survey had responses from 57,677 individuals in 15 municipalities (response rate, 70.9%). Of them, 52,320 individuals were linked with data on disability onset and mortality (follow-up rate, 90.7%). Excluding participants with a limitation in basic activities of daily living and those with invalid responses on age or gender, the final analysis sample comprised 48,474 individuals with 416,779 person-year observations. Their participation pattern in questionnaire surveys is shown in eTable 1.

Assessment of socioeconomic status and principal component analysis

The exposure, socioeconomic status (SES) at baseline, was assessed with equivalised household income including pension, equivalised household wealth, and respondents' years of education with the questionnaires in the 2013 survey. Household income was assessed with the following question: *"What was your pretax annual household net income in the past year (including pension)?"* with the response options of: <0.5, 0.5–0.9, 1.0–1.4, 1.5–2.0, 2.0–2.4, 2.5–2.9, 3.0–3.9, 4.0–4.9, 5.0–5.9, 6.0–6.9, 7.0–7.9, 8.0–8.9, 9.0–9.9, 10.0–11.9, and ≥12.0 million Japanese yen (JPY). Household wealth was assessed with the following question: *"What is the total value of your household assets, including savings, real estate (e.g. house, land, condominium), stocks, golf membership, etc.?"* with the response options of: <0.5, 0.5–0.9, 1.0–4.9, 5.0–9.9, 10.0–49.9, and ≥50.0 million JPY. The midpoints of brackets were assigned and equivalised by dividing by a square root of the number of household members. For the lowest and highest income categories, 2.5 and 13.0 were assigned. For the lowest and highest wealth categories, 2.5 and 50.0 were assigned. Years of education were responded

with the following options: <6 (coded 3), 6–9 (coded 7.5), 9–12 (coded 11), ≥12 (coded 16), and others (coded missing and imputed).

Principal component analysis was performed to incorporate the three dimensions of SES. The parallel analysis indicated that the first component to be retained, which also met the Kaiser-Guttman criteria (eFigure 2). The factor loading for SES and predicted factor scores are reported in eTable 2 and eFigure 3.

Selection, assessment, and cutoff values of mediator variables

The mediator variables were selected based on a previous study that reported the ten leading causes of total years lived with disability worldwide.[1] Considering relevant items included in the Japan Gerontological Evaluation Study survey, the following factors were selected: the number of natural teeth; depressive symptoms; subjective cognitive complaints; diabetes; stroke; eye disease; ear disease; falling experience. In addition, smoking status, drinking status, and walking time were added as traditional health behaviours.

The number of natural teeth was answered with the options of 0, 1–4, 5–9, 10–19, and ≥20 teeth; this variable was dichotomised to indicate having <20 or ≥20 teeth.[2] Depressive symptoms were assessed with the Geriatric Depression Scale 15, with a cutoff of the score ≥5 indicating having moderate depression.[3] Subjective cognitive complaints were assessed with three yes/no questions about subject memory complaints in the Kihon Checklist, a scale commonly used throughout Japan to screen older adults with high risk for frailty primarily. The subjective cognitive complaints score originally ranges from 0 to 3, and a score of ≥1 predicts the incidence of dementia.[4] The comorbid conditions were assessed by asking participants to choose diseases or health conditions under treatment or having long-term effects, including ear disease, diabetes, eye disease, and stroke. Falling experience in the past year was assessed with the response options of “many times,” “once,” and “none,” and

this variable was dichotomised to indicate having falling experience in the past year or not. Smoking status was answered with the options of "smoke," "smoked in the past," "never smoked" in 2013 and "smoke almost every day," "sometimes smoke," "quit smoking less than five years ago," "quit smoking more than five years ago," and "never smoked" in 2016 and 2019. This variable was dichotomised to indicate current smoking. Alcohol drinking status was answered with options of "drink alcohol," "drank alcohol in the past," "never drank" in 2013, and "drink alcohol," "quit drinking less than five years ago," "quit drinking more than five years ago," and "never drank" in 2016 and 2019. This variable was dichotomised to indicate current drinking. Physical activity was assessed with the average daily walking time with the options of <0.5, 0.5–1.0, 1.0–1.5, and ≥1.5 hours. This variable was dichotomised to indicate walking <0.5 or ≥0.5 hours per day following a previous study.[5]

The cutoffs for variables with multiple categories were determined based on previous literature. We also employed the Karlson–Holm–Breen (KHB) method with a single mediator to evaluate the mediating effect according to each cutoff and used the one exhibiting the largest indirect effect for each mediator.

To validate the variable of having eye or ear diseases, we compared the responses on visual and hearing impairments with visual and hearing impairment assessed with a 5-point Likert scale. This analysis was conducted using data of a subset of the study participants since the Japan Gerontological Evaluation Study surveys in 2016 and 2019 had eight different versions of the questionnaire and randomly selected one-in-eight participants answered the version including questions on visual and hearing impairment assessed with a 5-point Likert scale. We confirmed that having eye or ear diseases were strongly associated with vision or hearing impairment (eTable 3).

Discrete-time survival analysis

The data for discrete-time survival analysis was constructed in a long format, with one observation for every year of age in which the individual was a part of the study. Information on mediators, each measured at three time points, was organized into a single column based on participant age to estimate the mediator-specific indirect effect during the follow-up period. Accordingly, individuals who developed the outcome before each follow up survey does not have corresponding rows representing that survey. A complementary log-log regression model was fitted to approximate the Cox proportional hazards model. Both a crude model and a model adjusted for baseline confounders were estimated.

The KHB method

We employed the KHB method [6] to disentangle the association of SES with the onset of disability and mortality into indirect effects through each mediator and direct effects not through any of them. The KHB method first fits a nonlinear probability model, which was a complementary log-log regression in the present study, of the outcome with the exposure (ie, SES) adjusting for all mediators and confounders (ie, full model). Then, linear probability models were fitted for each mediator with the exposure and confounders (ie, mediator models). The residuals from the mediator models were then included in the outcome model with the exposure and all confounders but without mediators (ie, reduced model). Thereby, the difference in the coefficients of exposure in the reduced and full models indicates the joint indirect effect for all mediators, separated from the rescaling effect of nonlinear probability models. The joint indirect effect is further decomposed into paths through each mediator, that is, the product of the coefficient of SES for each mediator in the mediator models and the coefficient of the corresponding mediator for the outcome in the full model.

Multiple imputation procedure

Missing information on variables was imputed by multiple imputations with chained equations. Imputation diagnostics were evaluated by visual inspection of trace plots and comparing distributions of the imputed and observed variables. Ten imputed datasets for main analysis and five datasets for sensitivity analyses were created, and estimates were combined with Rubin's formula. The proportion of missing information ranged between 0% (age and gender) and 55.1% (GDS score in 2019) (eTable 4). Complete case participants had higher SES than the response sample. The imputed sample had similar demographic characteristics to the response sample, suggesting the selection bias was reduced (eTable 5).

References

1. GBD 2019 Ageing Collaborators. Global, regional, and national burden of diseases and injuries for adults 70 years and older: systematic analysis for the Global Burden of Disease 2019 Study. BMJ. 2022 Mar 10;e068208.
2. Petersen, P. E., Baez, R. J., & World Health Organization. Oral Health Surveys: Basic Methods. World Health Organization, Geneva, 2013.
3. Shin, C., Park, M. H., Lee, S.-H., Ko, Y.-H., Kim, Y.-K., Han, K.-M., Jeong, H.-G., & Han, C. Usefulness of the 15-item geriatric depression scale (GDS-15) for classifying minor and major depressive disorders among community-dwelling elders. Journal of Affective Disorders. 2019 259:370–375.
4. Tomata Y, Sugiyama K, Kaiho Y, Sugawara Y, Hozawa A, Tsuji I. Predictive ability of a simple subjective memory complaints scale for incident dementia: Evaluation of Japan's national checklist, the "Kihon Checklist." Geriatr Gerontol Int. 2017 Sep;17(9):1300–5.
5. Matsuyama S, Murakami Y, Lu Y, Sugawara Y, Tsuji I. Changes in time spent walking and disability-free life expectancy in Japanese older people: The Ohsaki Cohort 2006 Study. Prev Med. 2022 Oct;163:107190.

6. Breen R, Karlson KB, Holm A. Total, Direct, and Indirect Effects in Logit and Probit Models. Sociol Methods Res. 2013 May 1;42(2):164–91.

eTable 1. Participation pattern

Questionnaire survey wave				
2013	2016	2019	n[a]	%
R	R	R	19,132	39.5%
R	R	NR	10,945	22.6%
R	R	FE	2,976	6.1%
R	NR	R	2,035	4.2%
R	NR	NR	7,129	14.7%
R	NR	FE	2,678	5.5%
R	FE	FE	3,579	7.4%

FE, follow-up ended due to death, disability onset, or moving out; NR, not responded; R, responded.
[a] Number of participants linked with data from the municipal and national registry on the date of disability onset, all-cause mortality, or moving out.

eFigure 1. Flowchart of the study participants

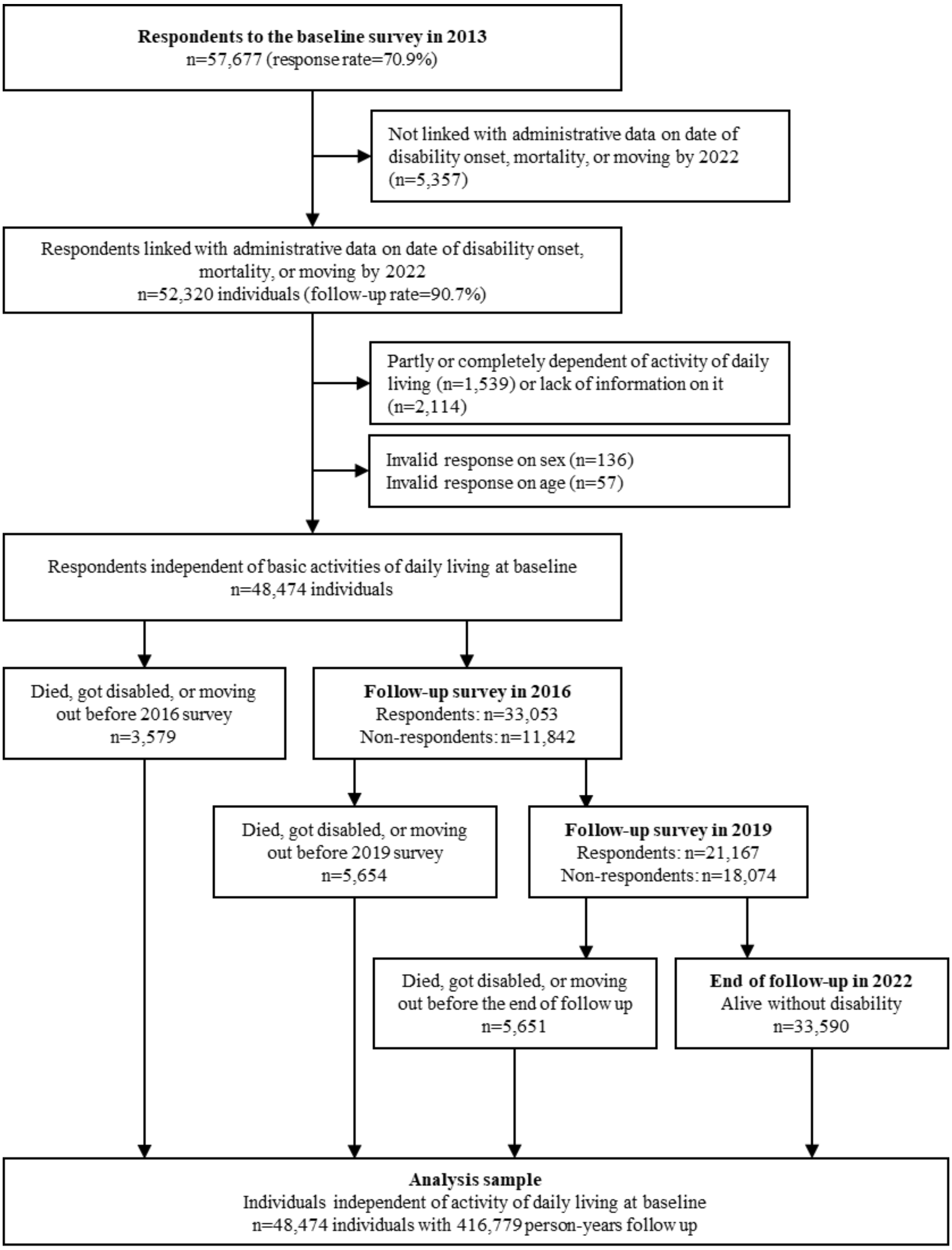

eFigure 2. Scree plot of eigenvalues from PCA analysis

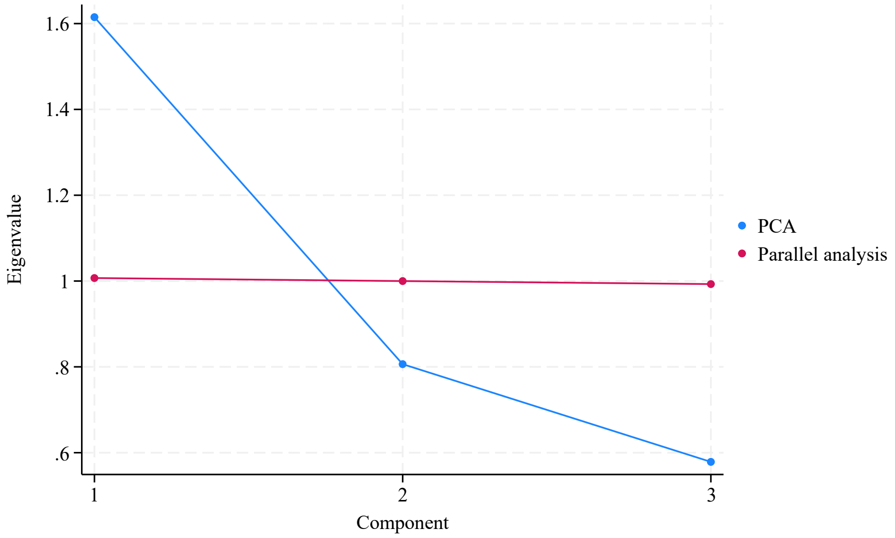

eTable 2. Factor loadings for socioeconomic status (n=48,474)

	Factor loading [a]
	Principal component 1
Socioeconomic status measurements	
Equivalised income	0.612
Equivalised wealth	0.621
Years of education	0.490

JPY, Japanese yen.

[a] Mean of factor loadings across the ten imputed datasets is reported.

eFigure 3. Histogram of factor score for socioeconomic status

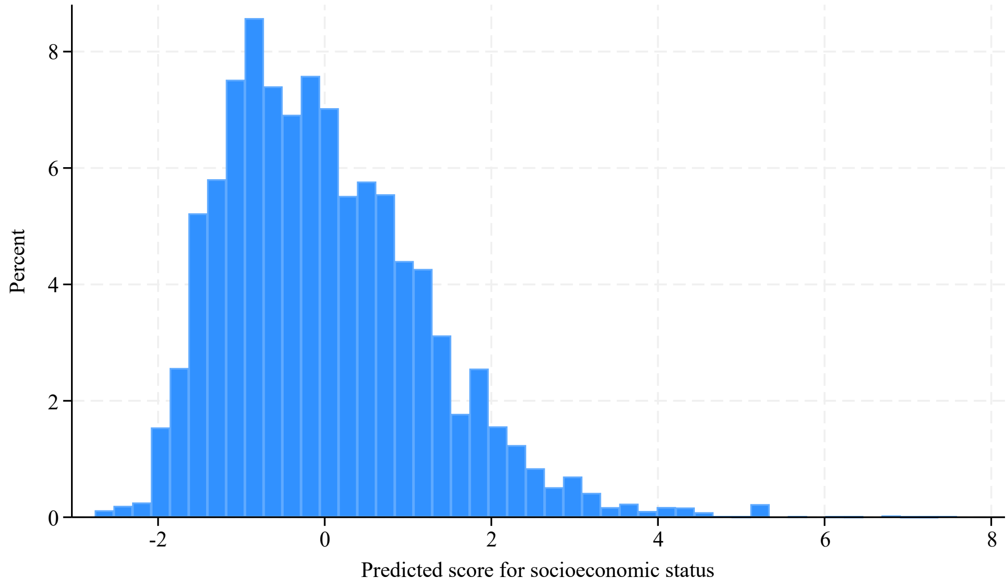

eTable 3. Association of having eye or ear disease with vision or hearing impairment: result from subsample responded to a particular version of the questionnaire in 2016 and 2019

	Having eye disease in 2016		
	No	Yes	
	n (%)	n (%)	P-value
Vision ability 2016			<0.001
Excellent	275 (91.4%)	26 (8.6%)	
Very good	866 (85.3%)	149 (14.7%)	
Good	1,608 (77.9%)	456 (22.1%)	
Fair	173 (48.1%)	187 (51.9%)	
Poor	3 (23.1%)	10 (76.9%)	

	Having ear disease in 2016		
	No	Yes	
	n (%)	n (%)	P-value
Hearing ability 2016			<0.001
Excellent	659 (99.5%)	3 (0.5%)	
Very good	991 (97.8%)	22 (2.2%)	
Good	1,362 (94.8%)	75 (5.2%)	
Fair	468 (80.1%)	116 (19.9%)	
Poor	20 (62.5%)	12 (37.5%)	

	Having eye disease in 2019		
	No	Yes	
	n (%)	n (%)	P-value
Vision ability 2019			<0.001
Excellent	105 (84.7%)	19 (15.3%)	
Very good	416 (83.4%)	83 (16.6%)	
Good	707 (74.1%)	247 (25.9%)	
Fair	97 (52.4%)	88 (47.6%)	
Poor	0 (0.0%)	3 (100.0%)	

	Having ear disease in 2019		
	No	Yes	
	n (%)	n (%)	P-value
Hearing ability 2019			<0.001
Excellent	268 (99.6%)	1 (0.4%)	
Very good	472 (97.1%)	14 (2.9%)	
Good	641 (94.1%)	40 (5.9%)	
Fair	226 (76.6%)	69 (23.4%)	
Poor	9 (60.0%)	6 (40.0%)	

eTable 4. Frequency of missing responses

	Analysis sample<br>n	Missing information<br>n (%)
**Variables in the 2013 survey**		
Equivalised income	48,474	9,228 (19.0%)
Equivalised wealth	48,474	13,784 (28.4%)
Years of education	48,474	942 (1.9%)
Age	48,474	0
Sex	48,474	0
Marital status	48,474	1,091 (2.3%)
Self-rated health	48,474	662 (1.4%)
Functional limitation [a]	48,474	3,172 (6.5%)
Having <20 teeth	48,474	1,302 (2.7%)
Depressive symptoms [b]	48,474	7,954 (16.4%)
Subjective cognitive complaints	48,474	1,132 (2.3%)
Diabetes	48,474	2,693 (5.6%)
Stroke	48,474	2,693 (5.6%)
Falling experience	48,474	662 (1.4%)
Eye disease	48,474	2,693 (5.6%)
Ear disease	48,474	2,693 (5.6%)
Current smoking	48,474	718 (1.5%)
Current drinking	48,474	634 (1.3%)
Walking time	48,474	914 (1.9%)
**Variables in the 2016 survey**		
Having <20 teeth	44,895	12,910 (28.8%)
Depressive symptoms [b]	44,895	17,857 (39.8%)
Subjective cognitive complaints	44,895	12,712 (28.3%)
Diabetes	44,895	13,152 (29.3%)
Stroke	44,895	13,152 (29.3%)
Falling experience	44,895	12,495 (27.8%)
Eye disease	44,895	13,152 (29.3%)
Ear disease	44,895	13,152 (29.3%)
Current smoking	44,895	12,654 (28.2%)
Current drinking	44,895	12,954 (28.9%)
Walking time	44,895	12,858 (28.6%)
**Variables in the 2019 survey**		
Having <20 teeth	39,241	18,928 (48.2%)
Depressive symptoms [b]	39,241	21,638 (55.1%)
Subjective cognitive complaints	39,241	18,720 (47.7%)
Diabetes	39,241	19,018 (48.5%)
Stroke	39,241	19,018 (48.5%)
Falling experience	39,241	18,474 (47.1%)
Eye disease	39,241	19,018 (48.5%)
Ear disease	39,241	19,018 (48.5%)
Current smoking	39,241	18,562 (47.3%)
Current drinking	39,241	18,952 (48.3%)
Walking time	39,241	18,847 (48.0%)

[a] Assessed with Tokyo Metropolitan Institute of Gerontology Index of Competence Scale
[b] Assessed with Geriatric Depression Scale 15

eTable 5. Comparison of response, complete-case, and imputed samples

	Response sample n=48,474 n (%)	Complete-case sample n=11,311 n (%)	Imputed sample n=48,474 n (%)
**Endpoint of follow-up by 2022**			
Became disabled	9,110 (18.8%)	1,853 (16.4%)	9,110 (18.8%)
Died	9,530 (19.7%)	2,548 (22.5%)	9,530 (19.7%)
Became disabled or died [a]	14,118 (29.1%)	3,264 (28.9%)	14,118 (29.1%)
**Socioeconomic status in 2013**			
Equivalent income (M JPY) [b]	2.3 (1.5)	2.5 (1.5)	2.2 (1.4)
Equivalent wealth (M JPY) [b]	13.7 (11.2)	15.0 (11.1)	13.0 (10.8)
Years of education [a]	10.3 (3.2)	11.1 (3.2)	10.3 (3.2)
**Baseline confounders in 2013**			
Age	73.6 (6.1)	72.6 (5.8)	73.6 (6.1)
Sex			
Men	22,586 (46.6%)	6,648 (58.8%)	22,586 (46.6%)
Women	25,888 (53.4%)	4,663 (41.2%)	25,888 (53.4%)
Marital status			
Not married	12,421 (26.2%)	2,233 (19.7%)	12,819 (26.4%)
Married	34,962 (73.8%)	9,078 (80.3%)	35,655 (73.6%)
Self-rated health			
Very good/good	39,809 (83.3%)	9,589 (84.8%)	40,344 (83.2%)
Poor/very poor	8,003 (16.7%)	1,722 (15.2%)	8,130 (16.8%)
Functional limitation [c]			
No	19,774 (43.6%)	5,333 (47.1%)	20,920 (43.2%)
Yes	25,528 (56.4%)	5,978 (52.9%)	27,554 (56.8%)
**Time-varying mediators in 2013**			
Having <20 teeth	22,967 (48.7%)	4,755 (42.0%)	23,788 (49.1%)
Moderate depression [d]	10,215 (25.2%)	2,536 (22.4%)	12,572 (25.9%)
Cognitive complaints [e]	16,511 (34.9%)	3,428 (30.3%)	16,959 (35.0%)
Diabetes	6,209 (13.6%)	1,552 (13.7%)	6,394 (13.2%)
Stroke	1,446 (3.2%)	329 (2.9%)	1,497 (3.1%)
Falling experience	10,760 (22.5%)	2,184 (19.3%)	10,946 (22.6%)
Eye disease	9,758 (21.3%)	2,286 (20.2%)	10,187 (21.0%)
Ear disease	2,907 (6.3%)	626 (5.5%)	3,033 (6.3%)
Current smoking	4,720 (9.9%)	1,199 (10.6%)	4,782 (9.9%)
Current drinking	17,166 (35.9%)	4,893 (43.3%)	17,358 (35.8%)
Less walking	11,892 (25.0%)	2,599 (23.0%)	12,160 (25.1%)
**Time-varying mediators in 2016**			
Having <20 teeth	15,389 (48.1%)	4,010 (41.7%)	22,931 (51.1%)
Moderate depression [d]	5,788 (21.4%)	1,798 (18.7%)	10,951 (24.4%)
Cognitive complaints [e]	11,255 (35.0%)	2,904 (30.2%)	16,548 (36.9%)
Diabetes	4,288 (13.5%)	1,391 (14.5%)	6,144 (13.7%)
Stroke	995 (3.1%)	283 (2.9%)	1,502 (3.3%)
Falling experience	7,933 (24.5%)	1,999 (20.8%)	11,657 (26.0%)
Eye disease	7,074 (22.3%)	2,058 (21.4%)	10,175 (22.7%)
Ear disease	2,017 (6.4%)	516 (5.4%)	2,983 (6.6%)
Current smoking	2,724 (8.4%)	916 (9.5%)	4,021 (9.0%)
Current drinking	11,427 (35.8%)	4,181 (43.5%)	15,589 (34.7%)
Less walking	9,127 (28.5%)	2,460 (25.6%)	13,642 (30.4%)
**Time-varying mediators in 2019**			
Having <20 teeth	9,833 (48.4%)	3,642 (43.3%)	20,527 (52.3%)

Moderate depression [d]	3,988 (22.7%)	1,681 (20.0%)	10,480 (26.7%)
Cognitive complaints [e]	7,093 (34.6%)	2,563 (30.5%)	14,864 (37.9%)
Diabetes	2,852 (14.1%)	1,271 (15.1%)	5,641 (14.4%)
Stroke	648 (3.2%)	257 (3.1%)	1,349 (3.4%)
Falling experience	5,186 (25.0%)	1,854 (22.0%)	10,700 (27.3%)
Eye disease	4,832 (23.9%)	1,989 (23.6%)	9,537 (24.3%)
Ear disease	1,468 (7.3%)	571 (6.8%)	3,139 (8.0%)
Current smoking	1,385 (6.7%)	629 (7.5%)	2,788 (7.1%)
Current drinking	7,255 (35.8%)	3,518 (41.8%)	12,909 (32.9%)
Less walking	6,135 (30.1%)	2,332 (27.7%)	13,156 (33.5%)

[a] Either of disability or all-cause mortality onset
[b] Values show mean and standard deviation.
[c] Assessed with the Tokyo Metropolitan Institute of Gerontology Index of Competence.
[d] Assessed with the Geriatric Depression Scale 15 score ≥5.
[e] Having subjective cognitive complaints ≥1.

eFigure 4. Directed acyclic graph

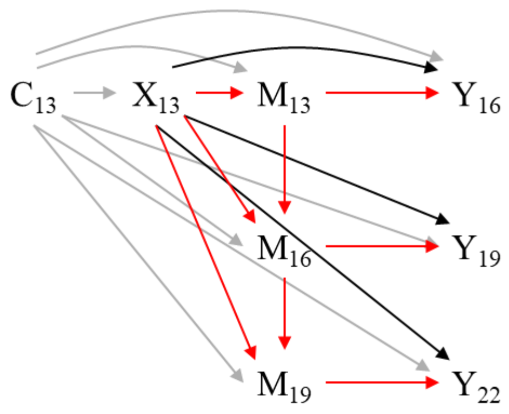

C: Baseline confounders, i.e., age, sex, marital status, self-rated health, functional limitation, and residential municipality
X: Exposure, i.e., socioeconomic status
M: Mediators, i.e., number of teeth, depression, diabetes, stroke, cognitive complaints, falling experience, eye disease, ear disease, smoking, drinking and walking time
Y: Outcome, i.e., disability and all-cause mortality
Subscripts: Survey wave
Black arrow lines: Direct effect paths
Red arrow lines: Indirect effect paths
Gray arrow lines: Confounding paths

eTable 6. Discrete-time survival analysis with complete case participants (n=92,460 person-year observations from 11,311 individuals)

	Incidence rate [a]	Crude HR (95% CI)	Adjusted HR (95% CI) [b]
SES quartile categories			
Highest	28.7	Reference	Reference
Higher	32.0	1.12 (1.00–1.24)	1.10 (0.98–1.22)
Lower	39.7	1.38 (1.25–1.53)	1.22 (1.10–1.35)
Lowest	57.9	2.00 (1.81–2.21)	1.54 (1.39–1.71)

CI, confidence interval; HR, hazard ratio; SES, socioeconomic status.

[a] Incidence rate per 1,000 person-years

[b] Adjusted for age, gender, marital status, self-rated health, and limitation in functional capacity, and residential municipality at the baseline.

eTable 7. Effect decomposition analysis with missing not at random assumption

	MNAR assumption: -5% point lower depression prevalence for missing data (delta = -0.25)			MNAR assumption: +5% point higher depression prevalence for missing data (delta = +0.25)		
	HR (95% CI)	*P*-value	PM, %	HR (95% CI)	*P*-value	PM, %
Total effect	1.098 (1.077–1.119)	<0.001	-	1.097 (1.076–1.117)	<0.001	-
Direct effect	1.041 (1.021–1.061)	<0.001	-	1.036 (1.015–1.057)	<0.001	-
Joint indirect effect	1.055 (1.049–1.060)	<0.001	57.0	1.059 (1.052–1.065)	<0.001	61.8
Having <20 teeth	1.012 (1.009–1.016)	<0.001	13.3	1.012 (1.009–1.016)	<0.001	13.3
Moderate depression [a]	1.013 (1.010–1.017)	<0.001	14.2	1.017 (1.013–1.022)	<0.001	18.7
Cognitive complaints [b]	1.006 (1.004–1.008)	<0.001	6.7	1.006 (1.004–1.008)	<0.001	6.7
Diabetes	1.000 (1.000–1.001)	0.107	0.4	1.000 (1.000–1.001)	0.106	0.4
Stroke	1.000 (1.000–1.001)	0.063	0.5	1.000 (1.000–1.001)	0.051	0.5
Falling experience	1.004 (1.003–1.005)	<0.001	3.8	1.004 (1.003–1.005)	<0.001	3.9
Eye disease	1.000 (1.000–1.001)	0.276	0.4	1.000 (1.000–1.001)	0.289	0.4
Ear disease	0.999 (0.999–1.000)	0.017	-0.7	0.999 (0.999–1.000)	0.013	-0.8
Current smoking	1.005 (1.003–1.006)	<0.001	4.9	1.005 (1.003–1.006)	<0.001	5.0
Current drinking	1.005 (1.003–1.007)	<0.001	5.4	1.005 (1.003–1.006)	<0.001	5.4
Less walking [c]	1.008 (1.006–1.009)	<0.001	8.2	1.008 (1.006–1.009)	<0.001	8.3

	MNAR assumption: -5% point lower tooth loss prevalence for missing data (delta = -0.25)			MNAR assumption: +5% point higher depression prevalence for missing data (delta = +0.25)		
	HR (95% CI)	*P*-value	PM–%	HR (95% CI)	*P*-value	PM, %
Total effect	1.097 (1.077–1.118)	<0.001	-	1.097 (1.077–1.118)	<0.001	-
Direct effect	1.040 (1.020–1.061)	<0.001	-	1.037 (1.017–1.057)	<0.001	-
Joint indirect effect	1.055 (1.049–1.062)	<0.001	57.8	1.058 (1.052–1.065)	<0.001	61.0
Having <20 teeth	1.011 (1.007–1.014)	<0.001	11.7	1.014 (1.010–1.018)	<0.001	15.1
Moderate depression [a]	1.016 (1.011–1.020)	<0.001	16.7	1.016 (1.012–1.020)	<0.001	16.7
Cognitive complaints [b]	1.006 (1.004–1.008)	<0.001	6.6	1.006 (1.004–1.008)	<0.001	6.6
Diabetes	1.000 (1.000–1.001)	0.105	0.4	1.000 (1.000–1.001)	0.100	0.4
Stroke	1.000 (1.000–1.001)	0.058	0.5	1.000 (1.000–1.001)	0.059	0.5
Falling experience	1.004 (1.003–1.005)	<0.001	3.8	1.004 (1.003–1.004)	<0.001	3.8
Eye disease	1.000 (1.000–1.001)	0.273	0.4	1.000 (1.000–1.001)	0.262	0.4
Ear disease	0.999 (0.999–1.000)	0.013	-0.8	0.999 (0.999–1.000)	0.016	-0.8
Current smoking	1.005 (1.003–1.006)	<0.001	5.0	1.005 (1.003–1.006)	<0.001	4.9
Current drinking	1.005 (1.003–1.007)	<0.001	5.3	1.005 (1.003–1.007)	<0.001	5.3
Less walking [c]	1.008 (1.006–1.009)	<0.001	8.2	1.008 (1.006–1.009)	<0.001	8.2

CI, confidence interval; HR, hazard ratio; MNAR, missing not at random; PM, proportion mediated.
Delta values of -0.25 and +0.25 represent assumptions that the probability of having moderate depression or tooth loss is different from that in the observed data by about -5, and 5 percentage points, respectively, due to the MNAR component of the missing mechanism.
Models were adjusted for age, gender, marital status, self-rated health, functional limitation, and residential municipality at the baseline.
[a] Assessed with the Geriatric Depression Scale 15 score ≥5.
[b] Having subjective cognitive complaints ≥1.
[c] Walking <0.5 hour a day

eTable 8. Effect decomposition analysis with complete case participants (n=92,460 person-year observations from 11,311 individuals)

	HR (95% CI)	*P*-value	PM (95% CI [d]), %
Total effect	1.167 (1.121–1.215)	<0.001	-
Direct effect	1.114 (1.069–1.161)	<0.001	-
Joint indirect effect	1.048 (1.039–1.057)	<0.001	30.1 (21.9–40.4)
Each indirect effect			
Having <20 teeth	1.015 (1.010–1.020)	<0.001	9.6 (5.1–14.2)
Moderate depression [a]	1.009 (1.005–1.013)	<0.001	6.0 (3.5–9.5)
Cognitive complaints [b]	1.005 (1.003–1.008)	<0.001	3.4 (1.8–5.4)
Diabetes	1.000 (0.999–1.001)	0.990	0.0 (-0.8 to 0.5)
Stroke	1.000 (1.000–1.001)	0.163	0.3 (0.0–0.9)
Falling experience	1.002 (1.001–1.003)	0.005	1.3 (0.4–2.6)
Eye disease	1.001 (1.000–1.002)	0.244	0.4 (-0.3 to 1.3)
Ear disease	0.999 (0.998–1.000)	0.047	-0.8 (-1.8 to -0.1)
Current smoking	1.004 (1.001–1.006)	0.001	2.3 (1.0–4.3)
Current drinking	1.004 (1.002–1.007)	0.001	2.9 (1.3–5.1)
Less walking [c]	1.007 (1.004–1.010)	<0.001	4.6 (2.5–7.0)

CI, confidence interval; HR, hazard ratio; PM, proportion mediated.
Models were adjusted for age, sex, marital status, self-rated health, limitations in functional capacity, and residential municipality at the baseline.
[a] Assessed with the Geriatric Depression Scale 15 score ≥5.
[b] Having subjective cognitive complaints ≥1.
[c] Walking <0.5 hour a day
[d] 95% CI were obtained using bootstrap with 200 resamples.

eTable 9. Effect decomposition analysis with different cutoffs. Different cutoffs were used for tooth loss (edentulous or having ≥1 teeth) and depression (having severe depression or not); multiple imputation applied

	HR (95% CI)	*P*-value	PM (95% CI [d]), %
Total effect	1.094 (1.069–1.119)	<0.001	-
Direct effect	1.053 (1.028–1.079)	<0.001	-
Joint indirect effect	1.039 (1.034–1.043)	<0.001	42.2 (33.3–54.2)
Each indirect effect			
Edentulousness	1.003 (1.002–1.005)	<0.001	3.7 (2.2–5.7)
Severe depression [a]	1.005 (1.002–1.008)	0.001	5.5 (2.1–8.7)
Cognitive complaints [b]	1.008 (1.006–1.009)	<0.001	8.4 (6.1–11.7)
Diabetes	1.000 (1.000–1.001)	0.101	0.4 (0.0–1.0)
Stroke	1.000 (1.000–1.001)	0.095	0.4 (0.0–1.0)
Falling experience	1.004 (1.002–1.005)	<0.001	3.9 (2.6–5.4)
Eye disease	1.000 (1.000–1.001)	0.122	0.5 (0.0–1.1)
Ear disease	0.999 (0.999–1.000)	0.016	-0.7 (-1.3 to -0.3)
Current smoking	1.005 (1.003–1.006)	<0.001	5.3 (3.7–7.1)
Current drinking	1.005 (1.004–1.007)	<0.001	5.8 (4.1–8.0)
Less walking [c]	1.008 (1.006–1.010)	<0.001	9.1 (6.8–11.9)

CI, confidence interval; HR, hazard ratio; PM, proportion mediated.
Models were adjusted for age, sex, marital status, self-rated health, limitations in functional capacity, and municipality of residence at the baseline.
[a] Assessed with the Geriatric Depression Scale 15 score ≥10.
[b] Having subjective cognitive complaints ≥1.
[c] Walking <0.5 hours a day
[d] 95% CI were obtained using bootstrap with 200 resamples for each imputed dataset.

eTable 10. Discrete-time survival analysis with separate outcomes; n=48,474 individuals; multiple imputation applied

	Incidence rate [a]	Crude HR (95% CI)	Adjusted HR (95% CI) [b]
**Outcome: mortality**			
SES quartile categories			
Highest	18.6	Reference	Reference
Higher	21.7	1.16 (1.09–1.23)	1.09 (1.02–1.16)
Lower	25.3	1.36 (1.27–1.45)	1.18 (1.11–1.26)
Lowest	29.0	1.59 (1.49–1.68)	1.24 (1.17–1.32)
**Outcome: disability** [c]			
SES quartile categories			
Highest	17.4	Reference	Reference
Higher	21.2	1.22 (1.14–1.31)	1.11 (1.04–1.20)
Lower	25.8	1.50 (1.40–1.61)	1.21 (1.13–1.31)
Lowest	31.9	1.89 (1.77–2.01)	1.29 (1.20–1.39)
**Outcome: mild disability** [d]			
SES quartile categories			
Highest	23.8	Reference	Reference
Higher	28.3	1.19 (1.12–1.27)	1.09 (1.03–1.16)
Lower	34.2	1.46 (1.37–1.55)	1.19 (1.12–1.26)
Lowest	42.6	1.84 (1.73–1.94)	1.25 (1.18–1.34)

CI, confidence interval; HR, hazard ratio.

[a] Incidence rate per 1,000 person-years

[b] Adjusted for age, gender, marital status, self-rated health, limitation in functional capacity, and residential municipality at the baseline.

[c] Certification for long-term care need level 2 or higher

[d] Certification for long-term care need level 1 or higher

eTable 11. Effect decomposition analysis with separate outcomes; n=48,474 individuals; multiple imputation applied

	Outcome: Mortality			Outcome: Disability [a]			Outcome: Mild disability [b]		
	HR (95% CI)	*P*-value	PM (95% CI [f]), %	HR (95% CI)	*P*-value	PM (95% CI [f]), %	HR (95% CI)	*P*-value	PM (95% CI [f]), %
Total effect	1.084 (1.059–1.109)	<0.001	-	1.107 (1.079–1.135)	<0.001	-	1.093 (1.070–1.117)	<0.001	-
Direct effect	1.036 (1.013–1.061)	0.003	-	1.048 (1.020–1.076)	0.001	-	1.042 (1.019–1.066)	<0.001	-
Joint indirect effect	1.046 (1.039–1.052)	<0.001	55.7 (42.7–78.6)	1.057 (1.049–1.065)	<0.001	54.2 (41.3–71.6)	1.049 (1.043–1.055)	<0.001	54.0 (42.0–70.2)
Indirect effect via each mediator									
Having <20 teeth	1.012 (1.008–1.016)	<0.001	14.7 (9.6–22.5)	1.009 (1.004–1.013)	<0.001	8.7 (4.0–13.5)	1.006 (1.003–1.010)	<0.001	7.2 (3.1–11.5)
Moderate depression [c]	1.013 (1.009–1.016)	<0.001	15.9 (11.0–23.6)	1.016 (1.011–1.021)	<0.001	15.4 (10.5–22.2)	1.013 (1.010–1.017)	<0.001	15.0 (10.9–20.7)
Cognitive complaints [d]	1.003 (1.001–1.005)	0.003	3.5 (1.4–6.2)	1.009 (1.007–1.011)	<0.001	8.8 (6.2–12.0)	1.010 (1.008–1.012)	<0.001	10.8 (8.0–14.6)
Diabetes	1.000 (1.000–1.001)	0.090	0.6 (0.0–1.5)	1.000 (1.000–1.001)	0.138	0.4 (0.0–1.0)	1.000 (1.000–1.001)	0.120	0.5 (0.0–1.2)
Stroke	1.000 (1.000–1.001)	0.052	0.5 (0.1–1.1)	1.001 (1.000–1.001)	0.087	0.5 (0.0–1.2)	1.001 (1.000–1.001)	0.080	0.6 (0.0–1.4)
Falling experience	1.002 (1.001–1.003)	<0.001	2.5 (1.4–4.1)	1.004 (1.003–1.005)	<0.001	4.1 (2.7–5.9)	1.004 (1.002–1.005)	<0.001	4.0 (2.6–5.8)
Eye disease	1.000 (1.000–1.001)	0.151	0.5 (-0.1 to 1.2)	1.000 (1.000–1.001)	0.214	0.3 (-0.1 to 0.9)	1.000 (1.000–1.001)	0.212	0.3 (-0.1 to 0.9)
Ear disease	0.999 (0.998–1.000)	0.012	-1.1 (-2.2 to -0.4)	0.999 (0.999–1.000)	0.086	-0.5 (-1.2 to 0.0)	1.000 (0.999–1.000)	0.133	-0.4 (-1.0 to 0.1)
Current smoking	1.005 (1.003–1.006)	<0.001	5.8 (4.0–8.8)	1.004 (1.002–1.005)	<0.001	3.8 (2.4–5.7)	1.004 (1.002–1.005)	<0.001	4.1 (2.7–6.1)
Current drinking	1.005 (1.003–1.006)	<0.001	5.8 (3.9–9.0)	1.005 (1.003–1.006)	<0.001	4.5 (2.8–6.7)	1.004 (1.002–1.005)	<0.001	4.3 (2.6–6.4)
Less walking [e]	1.006 (1.004–1.007)	<0.001	7.3 (5.1–10.9)	1.008 (1.006–1.010)	<0.001	8.3 (6.0–11.5)	1.007 (1.005–1.008)	<0.001	7.6 (5.5–10.7)

CI, confidence interval; HR, hazard ratio; PM, proportion mediated.

Models were adjusted for age, gender, marital status, self-rated health, functional limitation, and residential municipality at the baseline.

[a] Certification for long-term care need level 2 or higher

[b] Certification for long-term care need level 1 or higher

[c] Assessed with the Geriatric Depression Scale 15 score ≥5.

[d] Having subjective cognitive complaints ≥1.

[e] Walking <0.5 hour a day

[f] 95% CI were obtained using bootstrap with 200 resamples for each imputed dataset.

eTable 12. Causal mediation analysis for tooth loss and depression

	Mediator: having <20 teeth		Mediator: moderate depression	
	Model 1 [d]	Model 2 [e]	Model 1 [f]	Model 2 [g]
	ERR [h] (95% CI)	ERR [h] (95% CI)	ERR [h] (95% CI)	ERR [h] (95% CI)
Mediator 2013 model [a]				
TE	0.082 (0.06–0.10)	0.082 (0.06–0.10)	0.072 (0.05–0.09)	0.072 (0.05–0.09)
NIE	0.014 (0.01–0.02)	0.014 (0.01–0.02)	0.004 (0.00–0.01)	0.004 (0.00–0.01)
NDE	0.068 (0.05–0.09)	0.068 (0.05–0.09)	0.068 (0.05–0.09)	0.068 (0.05–0.09)
PM, %	16.7 (10.8–22.5)	16.7 (10.8–22.5)	5.1 (0.7–9.5)	5.1 (0.7–9.5)
Mediator 2016 model [b]				
TE	0.065 (0.04–0.09)	0.055 (0.03–0.08)	0.060 (0.03–0.09)	0.060 (0.03–0.09)
NIE	0.012 (0.01–0.02)	0.004 (-0.00 to 0.01)	0.011 (0.01–0.02)	0.008 (0.00–0.01)
NDE	0.053 (0.03–0.08)	0.051 (0.02–0.08)	0.049 (0.02–0.08)	0.053 (0.03–0.08)
PM, %	18.7 (7.8–29.5)	7.2 (-1.0 to 16.0)	18.4 (6.1–30.6)	13.1 (3.6–22.7)
Mediator 2019 model [c]				
TE	0.062 (0.03–0.10)	0.050 (0.01–0.08)	0.067 (0.03–0.10)	0.067 (0.03–0.11)
NIE	0.009 (0.00–0.02)	<0.001 (-0.01 to 0.01)	0.022 (0.01–0.03)	0.015 (0.01–0.02)
NDE	0.053 (0.02–0.09)	0.050 (0.01–0.09)	0.046 (0.01–0.08)	0.052 (0.01–0.09)
PM, %	14.6 (-1.0 to 30.0)	0.1 (-15.0 to 15.0)	32.7 (8.8–56.5)	22.6 (5.3–40.0)

CI, confidence interval; ERR, excess relative risk; NDE, natural direct effect; NIE, natural indirect effect; PM, proportion mediated; TE, total effect.
Cox proportional hazard model for outcome regression and logistic regression model for mediator regression was fitted.
[a] n=48,474
[b] n=44,895
[c] n=39,241
[d] Adjusted for baseline confounders and mediators other than tooth loss
[e] Adjusted for baseline confounders, mediators other than tooth loss, and tooth loss measured in prior waves
[f] Adjusted for baseline confounders and mediators other than moderate depression
[g] Adjusted for baseline confounders, mediators other than moderate depression, and moderate depression measured in prior waves
[h] Based on a comparison between counterfactual outcomes for the standardized socioeconomic status scores of -0.5 and 0.5.

eTable 13. Test for interaction effects between socioeconomic status and mediators for disability or mortality; results from discrete-time survival analysis with multiple imputation

	Interaction term	
	Hazard ratio	*P*-value
Having <20 teeth x Highest SES	Reference	
Having <20 teeth x Higher SES	0.99	0.872
Having <20 teeth x Lower SES	0.97	0.633
Having <20 teeth x Lowest SES	0.95	0.434
Depression x Highest SES	Reference	
Depression x Higher SES	0.95	0.448
Depression x Lower SES	1.00	0.954
Depression x Lowest SES	0.94	0.387
Cognitive complaints x Highest SES	Reference	
Cognitive complaints x Higher SES	1.03	0.693
Cognitive complaints x Lower SES	1.02	0.751
Cognitive complaints x Lowest SES	1.02	0.784
Diabetes x Highest SES	Reference	
Diabetes x Higher SES	0.99	0.940
Diabetes x Lower SES	1.05	0.624
Diabetes x Lowest SES	1.08	0.334
Stroke x Highest SES	Reference	
Stroke x Higher SES	1.05	0.740
Stroke x Lower SES	1.21	0.163
Stroke x Lowest SES	1.33	0.028
Falling experience x Highest SES	Reference	
Falling experience x Higher SES	0.99	0.943
Falling experience x Lower SES	0.91	0.129
Falling experience x Lowest SES	0.90	0.109
Eye disease x Highest SES	Reference	
Eye disease x Higher SES	1.00	0.963
Eye disease x Lower SES	0.95	0.492
Eye disease x Lowest SES	0.90	0.126
Ear disease x Highest SES	Reference	
Ear disease x Higher SES	1.04	0.797
Ear disease x Lower SES	1.12	0.388
Ear disease x Lowest SES	1.12	0.322
Smoking x Highest SES	Reference	
Smoking x Higher SES	1.08	0.455
Smoking x Lower SES	1.05	0.647
Smoking x Lowest SES	0.98	0.855
Drinking x Highest SES	Reference	
Drinking x Higher SES	1.00	0.949
Drinking x Lower SES	1.09	0.160
Drinking x Lowest SES	1.07	0.224

Less walking x Highest SES	Reference	
Less walking x Higher SES	1.02	0.756
Less walking x Lower SES	0.98	0.806
Less walking x Lowest SES	0.96	0.567

SES, socioeconomic status.
Models were adjusted for age, gender, marital status, self-rated health, functional limitation, and residential municipality at the baseline.
SES was categorized into quartile categories.

eTable 14. Effect decomposition analysis, further controlling for denture use as a covariate; n=48,474 individuals; multiple imputation applied

	HR (95% CI)	*P*-value	PM (95% CI [d]), %
Total effect	1.098 (1.076–1.120)	<0.001	-
Direct effect	1.042 (1.021–1.062)	<0.001	-
Joint indirect effect	1.054 (1.046–1.062)	<0.001	56.4 (46.6–70.0)
Each indirect effect			
Having <20 teeth	1.010 (1.007–1.014)	<0.001	10.7 (8.6–16.0)
Moderate depression [a]	1.015 (1.012–1.019)	<0.001	16.2 (12.4–20.6)
Cognitive complaints [b]	1.007 (1.005–1.008)	<0.001	7.3 (5.3–9.6)
Diabetes	1.000 (1.000–1.001)	0.133	0.4 (0.0–0.9)
Stroke	1.000 (1.000–1.001)	0.145	0.4 (-0.1 to 0.9)
Falling experience	1.003 (1.002–1.004)	<0.001	3.5 (2.4–5.1)
Eye disease	1.000 (1.000–1.001)	0.263	0.4 (-0.1 to 1.1)
Ear disease	0.999 (0.999–1.000)	0.001	-0.8 (-1.4 to -0.3)
Current smoking	1.004 (1.003–1.006)	<0.001	4.5 (3.3–6.1)
Current drinking	1.005 (1.004–1.007)	<0.001	5.7 (4.1–7.9)
Less walking [c]	1.008 (1.006–1.009)	<0.001	8.0 (5.9–10.6)

CI, confidence interval; HR, hazard ratio; PM, proportion mediated.
Models were adjusted for age, gender, marital status, self-rated health, functional limitation, residential municipality at the baseline, and denture use in each wave.
[a] Assessed with the Geriatric Depression Scale 15 score ≥5.
[b] Having subjective cognitive complaints ≥1.
[c] Walking <0.5 hour a day
[d] 95% CI were obtained using bootstrap with 200 resamples for each imputed dataset.

eTable 15. Association between principal component factors and disability or mortality

	Disability or mortality			
	Model 1		Model 2	
	HR (95% CI)	*P*-value	HR (95% CI)	*P*-value
Component 1 [a] (unit: 1 SD)	1.104 (1.082–1.127)	<0.001	1.105 (1.082–1.128)	<0.001
Component 2 (unit: 1 SD)			0.962 (0.944–0.981)	<0.001
Component 3 (unit: 1 SD)			0.957 (0.939–0.976)	<0.001
R^2 value	0.097		0.098	

CI, confidence interval; HR, hazard ratio; SD, standard deviation.

Models were adjusted for age, sex, marital status, self-rated health, limitations in functional capacity, and residential municipality at the baseline.

[a] Component 1 was used as the socioeconomic status indicator in the main analysis.
